# Supplementary material for: Evaluation of Theoretical Frameworks to Detect Correlates of HPV Vaccination in the Midwest, US, Using Structural Equation Modeling
Source: Vaccines (Basel). 2023 Dec 15;11(12):1856. doi: 10.3390/vaccines11121856 (PMC10747226; doi:10.3390/vaccines11121856)
Supplement: Supplementary file 1 [file vaccines-11-01856-s001.zip › vaccines-2738817-supplementary.pdf]

Table S1. Covariances among residual terms related constructs and items within a construct for the IHT, HBT and TPB derived measurement models.

| IHT derived measurement model | Theory of Planned Behavior and Health Belief Theory derived measurement model |
|---|---|
| (L1 WITH L2; L1 WITH L3; L1 WITH L4; L3 WITH L4; L3 WITH L5; L3 WITH L6; L4 WITH L6; L5 WITH L6; L6 WITH L7) (L1A WITH L1B; L1A WITH L1C; L1A WITH L1D; L1B WITH L1D; L1B WITH L1C; L1D WITH L1E; L1G WITH L1H; L3C WITH L3D; L3A WITH L3C; L4A WITH L4B; L5A WITH L5C; L5A WITH L5D; L5B WITH L5C; L5B WITH L5D; L7A WITH L7B; L7A WITH L7C; L7D WITH L7E;). | (L1 WITH L2; L1 WITH L3; L1 WITH L4; L3 WITH L4; L3 WITH L5; L4 WITH L5; L5 WITH L6) (L1A WITH L1B; L1A WITH L1C; L1A WITH L1D; L1B WITH L1D; L1B WITH L1C; L1B WITH L1C; L1D WITH L1E; L1G WITH L1H; L2B WITH L2C; L2B WITH L2G; L2C WITH L2D; L2G WITH L2F; L2G WITH L2H; L3D WITH L3C; L3A WITH L3C; L4A WITH L4B; L5A WITH L5C; L5A WITH L5D; L5B WITH L5C; L5B WITH L5D) |

Table S2. Factors associated with the attitudes about HPV vaccination among teenagers and young adults in Midwest, US, March/April, 2023.

| **Exposure** | **Mediator** | **Outcome** | **β (95% CI)** |
|---|---|---|---|
| Beliefs about the benefits of vaccination | N.A. | Perceived benefits of HPV vaccination | 0.07 (-0.13, 0.26) |
| Beliefs about barriers to vaccination | N.A. | Perceived benefits of HPV vaccination | -0.27 (-0.41, -0.13) |
| Beliefs about susceptibility to HPV infection and cervical cancer | N.A. | Perceived benefits of HPV vaccination | **0.57 (0.24, 0.90)** |
| Beliefs about severity of HPV infection and cervical cancer | N.A. | Perceived benefits of HPV vaccination | **1.14 (0.87, 1.41)** |
| Beliefs about benefits of vaccination | N.A. | Perceived barriers to HPV vaccination | **0.72 (0.25, 1.19)** |
| Beliefs about barriers to vaccination | N.A. | Perceived barriers to HPV vaccination | **1.84 (0.99, 2.69)** |
| Beliefs about susceptibility to HPV infection and cervical cancer | N.A. | Perceived barriers to HPV vaccination | -0.03 (-0.29, 0.22) |
| Beliefs about severity of HPV infection and cervical cancer | N.A. | Perceived barriers to HPV vaccination | **-0.26 (-0.40, -0.11)** |
| Knowledge and awareness about HPV infection, cervical cancer and HPV vaccine | Beliefs about benefits of vaccination | Perceived benefits of HPV vaccination | 0.05 (-0.09, 0.18) |
| | Beliefs about barriers of vaccination | Perceived benefits of HPV vaccination | **0.15 (0.05, 0.25)** |
| | Beliefs about susceptibility to HPV infection and cervical cancer | Perceived benefits of HPV vaccination | -0.12 (-0.23, -0.01) |
| | Beliefs about severity of HPV infection and cervical cancer | Perceived benefits of HPV vaccination | **0.83 (0.61, 1.06)** |
| Total | | | **0.91 (0.82, 1.00)** |

| | | | |
|---|---|---|---|
| Knowledge and awareness about HPV infection, cervical cancer and HPV vaccine | Beliefs about benefits of vaccination | Perceived barriers to HPV vaccination | **0.50 (0.10, 0.90)** |
| | Beliefs about barriers of vaccination | Perceived barriers to HPV vaccination | **-1.03 (-1.44, -0.61)** |
| | Beliefs about susceptibility to HPV infection and cervical cancer | Perceived barriers to HPV vaccination | 0.07 (-0.06, 0.07) |
| | Beliefs about severity of HPV infection and cervical cancer | Perceived barriers to HPV vaccination | **-0.19 (-0.31, -0.06)** |
| Total | | | **-0.70 (-0.85, -0.56)** |
| Age | Beliefs about benefits of vaccination | Perceived benefits of HPV vaccination | 0.00 (-0.005, 0.006) |
| | Beliefs about barriers to vaccination | Perceived benefits of HPV vaccination | -0.005 (-0.02, 0.01) |
| | Beliefs about susceptibility to HPV infection and cervical cancer | Perceived benefits of HPV vaccination | **0.06 (0.003, 0.12)** |
| | Beliefs about severity of HPV infection and cervical cancer | Perceived benefits of HPV vaccination | 0.03 (-0.04, 0.11) |
| Total | | | **0.09 (0.01, 0.17)** |
| Age | Beliefs about benefits of vaccination | Perceived barriers to HPV vaccination | |
| | Beliefs about barriers to vaccination | Perceived barriers to HPV vaccination | 0.04 (-0.07, 0.14) |
| | Beliefs about susceptibility to HPV infection and cervical cancer | Perceived barriers to HPV vaccination | -0.003 (-0.04, 0.03) |
| | Beliefs about severity of HPV infection and cervical cancer | Perceived barriers to HPV vaccination | -0.008 (-0.02, 0.01) |
| Total | | | 0.03 (-0.05, 0.105) |

| | | | |
|---|---|---|---|
| Gender | Beliefs about benefits of vaccination | Perceived benefits of HPV vaccination | 0.004 (-0.01, 0.02) |
| | Beliefs about barriers to vaccination | Perceived benefits of HPV vaccination | 0.09 (-0.003, 0.02) |
| | Beliefs about susceptibility to HPV infection and cervical cancer | Perceived benefits of HPV vaccination | -0.03 (-0.07, 0.009) |
| | Beliefs about severity of HPV infection and cervical cancer | Perceived benefits of HPV vaccination | **0.11 (0.05, 0.18)** |
| Total | | | **0.09 (0.04, 0.15)** |
| Gender | Beliefs about benefits of vaccination | Perceived barriers to HPV vaccination | 0.05 (0.00, 0.10) |
| | Beliefs about barriers to vaccination | Perceived barriers to HPV vaccination | -0.06 (-0.13, 0.01) |
| | Beliefs about susceptibility to HPV infection and cervical cancer | Perceived barriers to HPV vaccination | 0.002 (-0.01, 0.02) |
| | Beliefs about severity of HPV infection and cervical cancer | Perceived barriers to HPV vaccination | **-0.03 (-0.04, -0.01)** |
| Total | | | -0.04 (-0.09, 0.02) |
| Educational status | Beliefs about benefits of vaccination | Perceived benefits of HPV vaccination | -0.003 (-0.01, 0.01) |
| | Beliefs about barriers to vaccination | Perceived benefits of HPV vaccination | -0.011 (-0.03, 0.005) |
| | Beliefs about susceptibility to HPV infection and cervical cancer | Perceived benefits of HPV vaccination | 0.04 (-0.07, 0.09) |
| | Beliefs about severity of HPV infection and cervical cancer | Perceived benefits of HPV vaccination | -0.01 (-0.09, 0.07) |
| Total | | | 0.02 (-0.06, 0.10) |
| Educational status | Beliefs about benefits of vaccination | Perceived barriers to HPV vaccination | -0.03 (-0.09, 0.03) |

| | | | |
|---|---|---|---|
| | Beliefs about barriers to vaccination | Perceived barriers to HPV vaccination | 0.08 (-0.03, 0.18) |
| | Beliefs about susceptibility to HPV infection and cervical cancer | Perceived barriers to HPV vaccination | -0.002 (-0.02, 0.02) |
| | Beliefs about severity of HPV infection and cervical cancer | Perceived barriers to HPV vaccination | 0.002 (-0.01, 0.02) |
| Total | | | 0.05 (-0.03, 0.13) |
| Ethnicity | Beliefs about benefits of vaccination | Perceived benefits of HPV vaccination | -0.001 (-0.005, 0.003) |
| | Beliefs about barriers to vaccination | Perceived benefits of HPV vaccination | -0.008 (-0.02, 0.002) |
| | Beliefs about susceptibility to HPV infection and cervical cancer | Perceived benefits of HPV vaccination | 0.01 (-0.02, 0.04) |
| | Beliefs about severity of HPV infection and cervical cancer | Perceived benefits of HPV vaccination | -0.049 (-0.10, 0.001) |
| Total | | | -0.05 (-0.11, 0.008) |
| Ethnicity | Beliefs about benefits of vaccination | Perceived barriers to HPV vaccination | -0.001 (-0.005, 0.004) |
| | Beliefs about barriers to vaccination | Perceived barriers to HPV vaccination | -0.008 (-0.02, 0.004) |
| | Beliefs about susceptibility to HPV infection and cervical cancer | Perceived barriers to HPV vaccination | 0.008 (-0.03, 0.04) |
| | Beliefs about severity of HPV infection and cervical cancer | Perceived barriers to HPV vaccination | -0.05 (-0.11, 0.008) |
| Total | | | **0.06 (0.001, 0.10)** |
